# Long Term Effects of the COVID-19 Pandemic on Social Concerns

**DOI:** 10.3389/fpsyg.2021.743054

**Published:** 2021-10-05

**Authors:** Esther Blanco, Alexandra Baier, Felix Holzmeister, Tarek Jaber-Lopez, Natalie Struwe

**Affiliations:** ^1^Department of Public Finance, University of Innsbruck, Innsbruck, Austria; ^2^The Ostrom Workshop, Indiana University, Bloomington, IL, United States; ^3^Department of Economics, University of Innsbruck, Innsbruck, Austria; ^4^Université Paris Nanterre, Nanterre, France

**Keywords:** charitable donation, COVID-19 pandemic, climate crisis, poverty, substitution of social concerns

## Abstract

While some local, temporary past crises have boosted overall charitable donations, there have been concerns about potential substitution effects that the Covid-19 pandemic might have on other social objectives, such as tackling climate change and reducing inequality. We present results from a donation experiment (*n* = 1, 762), with data collected between April 2020 and January 2021. We combine data from (i) an online donation experiment, (ii) an extended questionnaire including perceptions, actions, and motives on the Covid-19 pandemic, the climate crisis, and poverty, as well as charitable behavior and (iii) epidemiological data. The experimental results show that donations to diverse social concerns are partially substituted by donations to the Covid-19 fund; yet, this substitution does not fully replace all other social concerns. Over time we observe no systematic trend in charitable donations. In regards to the determinants of individual donations, we observe that women donate more, people taking actions against Covid-19 and against poverty donate more, while those fearing risks from poverty donate less. In addition, we observe that the population under consideration is sensitive to the needs of others, enhancing total donations for higher Covid-19 incidence. For donations to each charity, we find that trusting a given charitable organization is the strongest explanatory factor of donations.

**JEL:** L3, D64, Q54, I3, D9

## 1. Introduction

Understanding the drivers of human behavior is essential when facing global shocks such as the Covid-19 pandemic. Together with governmental actions and recommendations, the behavioral responses of citizens have shown to be a key variable in shaping the evolution of the collective action problem that the pandemic represents. A large body of literature has been dealing with the striking psychological consequences of the lockdown due to Covid-19 (see Salari et al., 2020, for an overview). Similarly, behavioral scientists have been tracking the evolution of social preferences and their correlation with health behaviors during the pandemic (see section 2 for a review). This study contributes to the literature addressing the long term (10 months) impact of the pandemic on social preferences by investigating the substitution effects in social concerns with respect to Covid-19, the climate crisis, and poverty alleviation. To the best of our knowledge, this is the first study to analyze whether the pandemic affects the social priorities during an extended time period.

Understanding the substitution in social concerns associated with the pandemic is critical in designing recovery policies. The relative weights of social concerns can affect the social acceptability of policies to “build back better.” Next to the substantial impact on individuals' daily lives and health conditions brought about by the Covid-19 pandemic, there are further pressing social objectives affecting human well-being, such as alleviating global poverty, addressing the climate crisis, and promoting environmental conservation (featured in the United Nations Sustainable Development Goals; SDGS). Importantly, these objectives are interrelated with the Covid-19 pandemic. The “Covid-19 Response” to each of the SDGs (United Nations, 2020) and the report by the World Wide Fund for Nature (WWF, Jeffries B., 2020) illustrate the complex interrelations between health, poverty, and environmental conservation. But these complex interrelationships might be difficult to perceive for citizens who since the beginning of the pandemic have been facing increased stress, burdens in their daily lives, and new economic challenges. This may translate into focusing on the pandemic at the expense of other pressing issues, substituting the relevance of previous social concerns. The apprehension of such substitution effects in social concerns induced by the Covid-19 has been stressed by researchers (see, e.g., Hodges and Jackson, 2020; Naidoo and Fisher, 2020), Think Tanks (see, e.g., Zhongming et al., 2020), and political leaders (such as those of the European Union (*EU*) early on). For example, Rosenbloom and Markard (2020) have raised the concern that the Covid-19 response and recovery could affect the mitigation of the climate crisis and the continuation of the *Intergovernmental Panel on Climate Change* (*IPCC*) report (Tollefson, 2020). In addition, Mahler et al. (2020) estimate that the Covid-19 pandemic might push about 40–60 million people into extreme poverty. Furthermore, a common concern of scientists, governments, and supra-national agencies is that the pandemic might induce a financial crisis amplifying inequality and severe poverty (von Braun et al., 2020).

Within this context, we present evidence on the long-term substitution effects that the Covid-19 pandemic might have on other social priorities by means of real-life donations to charities. We collected weekly data for 8 weeks and monthly data for 8 months between April 2020 and January 2021. Our results respond to the call by the scientific community for economists to contribute to the understanding of the behavioral effects of the Covid-19 pandemic (Coyle, 2020), contributing to the efforts by the economics discipline to generate cumulative evidence aiding policy-making (see https://bit.ly/3jmBZk3). We study if and how Covid-19 concerns substituted donations to other social concerns, how substitution effects evolved over time, and the determinants of donation behavior during the pandemic.

We combine results from (i) an online donation experiment with more than 1,700 students, (ii) an extended questionnaire, and (iii) epidemiological data. In the online donation experiment, subjects are endowed with €3 that can be distributed between themselves and a list of charitable organizations which vary between treatments. In a *Baseline* setting, the list of possible recipients comprises eight charities representing diverse social concerns. To measure potential substitution effects in donations between various social concerns in light of the Covid-19 pandemic, in a *COVID-19* treatment we include the *COVID-19 Solidarity Response Fund for WHO* (*WHO Covid-19 Fund*; see https://bit.ly/3wiwJDU for details about the fund) in addition to these eight charities as a possible recipient for donations. Finally, in a *Covid-19 Only* treatment we include only the *WHO Covid-19 Fund* as a possible recipient^1^. After the donation task, participants answer an extensive questionnaire including subjects' socio-demographic characteristics; subjects' perception of how relevant a charity's work is regarding alleviating the consequences of the Covid-19 pandemic, their national or international operation, and their trustworthiness; participants' risk perceptions, actions, and motivations regarding the Covid-19 pandemic, the climate crisis, and poverty, respectively; as well as subjects' history of donation and voluntary work for charities. Our pre-registered initial theses (see pre-registration at https://aspredicted.org/3g8sd.pdf) are (i) that the Covid-19 pandemic substitutes other social concerns, (ii) that the distribution of donations changes over time with the intensity of the crisis, and (iii) that donations correlate with risk perceptions, actions, and motives at the individual level. The controlled experiment that we present allows us to test these conjectures.

As compared to previous studies focusing on aggregate levels of charitable donations to single charities (see, e.g., Andreoni, 1990; Vesterlund, 2003; Frey and Meier, 2004; Bénabou and Tirole, 2006; Ariely et al., 2009; Gneezy et al., 2014; Garcia et al., 2020) or alternative charities with the same social objectives (see, among others, Soyer and Hogarth, 2011; Schmitz, 2021), we intentionally incorporate charities that cover a wide range of social priorities (as in Eckel and Grossman, 2003; Crumpler and Grossman, 2008; Brown et al., 2017). Our study is closer to previous contributions to the literature focusing on how negative shocks on individuals' health or natural disasters affect donations to charities working on related social objectives and to charities working on other social objectives (see section 2). Blanco et al. (2020) is the only previous experimental study looking at the effect of the pandemic on relative social priorities, reporting short time effects for 2 months. As compared to this study, we incorporate two main novelties: First, we present evidence for 10 months, providing evidence on long-term substitution of social objectives for the first time. Second, we provide a broad analysis on the individual determinants of donations to charities during the pandemic. The rich database collected through the questionnaire provides insights into the factors shaping human behavior in the context of the pandemic. We also incorporate the evolution of the epidemiological situation in the analysis of the determinants of donations (similar to other studies in this field of research, e.g., Abel et al., 2020; Branas-Garza et al., 2020; Lohmann et al., 2020).

Our findings suggest a long-term substitution effect due to the Covid-19 pandemic, as has been anticipated by policy makers. This result is derived from two observations: On the one hand, we observe substantial donations to the *WHO Covid-19 Fund*. On the other hand, participants do not change their aggregate donations depending on whether the *WHO Covid-19 Fund* is a possible recipient. These findings suggest that people react to the context and adapt their donation behavior to the broader set of calls for donations, but the aggregate social concern (altruism) is not reduced by the pandemic. The latter represents additional evidence on the mixed results in the literature with respect to the impact of the Covid-19 on social preferences (see section 2). Notably, we do not observe systematic trends in donations over time, which is possibly driven by the pandemic having extended over a longer time period than initial forecasts suggested. With the 10 months data collection in our analysis we have not, unfortunately, reached the post-pandemic period. We observe that systematic predictors of donation for the pooled data are the 7-day incidence of Covid-19 infections, self-reported individual Covid-19 actions, and participants' gender, with women donating significantly more than men, the latter being in line with previous literature (Eckel and Grossman, 2001, 2003; Eckel et al., 2005). When analyzing each organization separately, we find that trusting the corresponding charity is the most significant predictor of donations to the respective charity. This is in line with the emphasis of Ostrom (1990) on the relevance of trust as a precondition to successfully overcome collective action challenges.

## 2. Related Literature

Psychologists have devoted much effort during the Covid-19 pandemic to track the consequences of health regulations on psychological well-being (see Salari et al., 2020). At early stages of the Covid-19 pandemic, increased levels of depression, stress, and anxiety were reported for different populations (see Cao et al., 2020; Zhou et al., 2020 for college students, Wang and Zhao, 2020 for university students, and Zhang et al., 2020 for working adults in China; see Odriozola-Gonzalez et al., 2020a,b; Planchuelo-Gomez et al., 2020; Rodriguez-Rey et al., 2020 for evidence from different populations in Spain). Large scale studies have analyzed the early psychological responses to the pandemic, including concern and stress, and associated public behavior in 48 countries (Lieberoth et al., 2021). There is evidence that negative psychological effects endure for longer time periods (see, e.g., Gonzalez-Sanguino et al., 2020; Roma et al., 2020). More generally, life satisfaction (in a sample of Spanish adults) positively correlates with hope about overcoming the pandemic and negatively correlates with social phobia (Blasco-Belled et al., 2020). In addition, daily life satisfaction and the length of lockdown periods are positively correlated (Sabater-Grande et al., 2021).

Behavioral scientists have concurrently tracked the pro-social concerns during the pandemic. That is, the extent to which people care about others' well-being. Neoclassical economics commonly conceives individuals as purely self-interested decision makers, maximizing individual payoffs. Building on empirical evidence showing that individuals are similarly motivated by other-regarding preferences, such as altruism and inequality aversion supports a broader view on subjects' social preferences. In this study we focus on pro-social concerns (see Andreoni, 1989; Meier, 2007; Chaudhuri, 2011, for reviews on pro-social behavior). One way to elicit pro-social behavior is to look at donation decisions of individuals to charities. Next to looking at donation data of households from national statistics or survey measures, people's social concerns can be measured by means of experimental methods (see Levitt and List, 2007 for an overview of games used in experimental economics). A common approach to experimentally elicit prosocialty is to ask participants to decide on how to distribute a given amount of money between themselves and a charity recipient of their choice (Andreoni, 1990; Eckel and Grossman, 1996). Previous studies have identified factors systematically affecting subjects' pro-social behavior: Donation levels vary with the individual characteristics of donors (Eckel and Grossman, 2001) and the institutional context (Frey and Meier, 2004; Garcia et al., 2020), including whether there are market interactions (see, e.g., Bartling et al., 2015; Kirchler et al., 2016).

The stability of social concerns is a controversial topic. While several models characterize people as belonging to certain preference types (in the sense of latent traits), there is growing evidence that social concerns can be context-dependent, time-dependent, and vary with the experience of people in life. Individuals' pro-social preferences measured via experiments or surveys change over time due to factors like education interventions (Jakiela et al., 2015), economic shocks (Fisman et al., 2015), or natural disasters and violence (Voors et al., 2012; Cassar et al., 2017). Empirical studies using donation statistics show that fundraising interventions for natural and humanitarian disasters foster donations to charities related to the disaster, and increase donations to unrelated causes (Brown et al., 2012), but the effect on other charities fades out over time (Scharf et al., 2017). Importantly, such donations appeals have shown not to reduce donations to other (unrelated) causes (Deryugina and Marx, 2021). More specifically, Brown et al. (2012) show that unexpected donations of households after the 2004 Indian Ocean tsunami were positively correlated with planned (future) donations toward other social causes. Scharf et al. (2017) find that fundraising interventions associated with a natural or human disaster lift donations to charities related to the disaster, and donations to other (unrelated) charities for a short time but decline shortly thereafter, leading to no changes in baseline donation levels to the other charities in the longer time horizon. Similarly, Deryugina and Marx (2021) identify that an exogenous increase in demand for giving (due to tornadoes) does not reduce donations to other local charities. Thus, Deryugina and Marx (2021) conclude that “giving to one cause need not come at the expense of another.” An additional line of literature addresses the question whether and how experiencing a crisis affects peoples' pro-social behavior. For example, experiencing a natural disaster has been shown to reduce donations to related causes (Eckel et al., 2007); experiencing an adverse health shock (e.g., stroke, heart attack, cancer), however, substitutes donations to other social concerns toward health-related charities (Black et al., 2020). Our take from these studies measuring the effect of experience during a crisis is that the impact could be context dependent, and thus reinforces the need for specific research conducted for the Covid-19 pandemic.

Recent research on the effects of the Covid-19 pandemic on social preferences reports intertemporal stability of risk and time preferences (Drichoutis and Nayga, 2020) and a negative effect on generosity measured by donations in an online experiment (Branas-Garza et al., 2020). A study with students from Wuhan during the pandemic finds positive trends in altruism, trust, and risk tolerance (Shachat et al., 2020). Subjects in China that were more intensively exposed to the Covid-19 crisis reveal more anti-social behavior than those with lower exposure (Lohmann et al., 2020). Li et al. (2021) conducted an online experiment to examine the contagion of others' positive and negative donation behavior of the Covid-19 pandemic in China during and after the peak. They also investigated the impact of social anxiety on the link between the contagion of donation behaviors and the changes in the Covid-19 situation. Their results show that increased or decreased donation amounts given by other participants lead to positive or negative donation behavior, respectively. Moreover, participants' social anxiety decreased with the ease of the pandemic, and social anxiety in turn mediated the relationship between the pandemic abatement and the decrease in the contagion of positive donation behaviors.

Similarly, recent studies address how experience with the Covid-19 pandemic (Branas-Garza et al., 2020; Shachat et al., 2020) or information policies on Covid-19 affect people's pro-social behavior and pro-conservation policy support (Abel and Brown, 2020; Abel et al., 2020; Guo et al., 2020; Shreedhar and Mourato, 2020). Other studies have addressed, more broadly, the interconnections between the Covid-19 pandemic, economic well-being, and environmental conservation (see, e.g., Dobson et al., 2020; Goldthau and Hughes, 2020). Although a negative income shock due to the pandemic might decrease pro-social behavior (Almunia et al., 2020), previous evidence suggests that a collective threat can enhance cooperation, pro-social behavior, and trust (Li et al., 2020). Examining social preferences in the time of a pandemic is of special interest, as measures of social preferences have been found to correlate with health behavior. For example, people who are more pro-social are also more likely to follow hygiene recommendations to fight the pandemic (Campos-Mercade et al., 2021).

This study is a follow-up study of Blanco et al. (2020), which is—to the best of our knowledge—the first to report evidence on substitution effects between social concerns in the Covid-19 context. Blanco et al. (2020) investigate short-time changes in social concerns at the onset of the pandemic^2^. The results of Blanco et al. (2020) show a partial substitution of donations to a Covid-19-related fund at the expense of donations to other social concerns on the short run. The follow-up study presented herein is novel in two aspects: First, we examine long-time trends in social concerns, reporting data over 10 months. Second, we explore a wide set of determinants that might influence the donation behavior during the pandemic.

This study also contributes to a strand of literature investigating competition among charities, including studies using lab and field experiments. There is evidence from laboratory experiments that the total amount of charitable giving varies when changing the number of charities or campaigns (Reinstein, 2007; Soyer and Hogarth, 2011; Deck and Murphy, 2019; Schmitz, 2021), when the number of potential charities is uncertain (Eckel et al., 2020). Specifically, studies show that increasing the number of charities with similar objectives that are possible recipients increases total contributions (Soyer and Hogarth, 2011; Schmitz, 2021). Schmitz (2021) increases the list of charities from one single charity up to three and finds a weak substitution with more recipients but no changes in the overall donation amount. Soyer and Hogarth (2011) investigate competition among charities with up to 16 possible recipients. They show that the total amount of donations increases with more recipients but at a decreasing rate. There is also field evidence pointing in the same direction: A solicitation of volunteering by two charities results in increased time donations to each charity as compared to people solicited by a single charity to volunteer (Lange and Stocking, 2012). Lange and Stocking (2012) also show that subjects solicited to volunteer by two charities gave higher total monetary donations to the sum of charities than they gave when they were solicited by only one charity^3^.

In sum, the evidence from the studies discussed above suggests that donations to unexpected events caused by crises do not necessarily come at the expense of donations to other charities. When people have experienced the respective events themselves, the results seem to be context dependent: There is evidence that having experienced a health shock can generate a shift in donations, leading to a substitution toward donations to health related charities at the expense of donations toward other social concerns. This calls for specific results referring to the effects of the Covid-19 pandemic. While there is a wide literature on the effect of the pandemic on psychological well-being and social preferences, there is no study investigating the long-term substitution effects on social preferences that we address in this paper. Lastly, from a methodological perspective, the previous literature suggests that increasing the number of possible recipients increases total donations.

## 3. Experimental Design

We implement three different treatment conditions, where each of the three treatments consists of a incentivized donation-to-charity task, similar to Eckel and Grossman (2003) and Eckel et al. (2005), followed by a questionnaire. In the donation task, subjects were endowed with €3 to be distributed among themselves and various charitable organizations, freely deciding how much to allocate to each charity and to themselves, if any. The list of available charities varied between treatments.

In the *Baseline* treatment, the list of charitable organizations included eight charities, namely *World Wide Fund for Nature* (*WWF*), *Doctors Without Borders* (*MSF*), *Amnesty International* (*AI*), *SOS Kinderdorf* (*SOS*), *Caritas* (*CAR*), *Licht ins Dunkel* (*LID*), *Oxfam* (*OXF*), and the *Red Cross* (*RC*). This list was chosen to reflect a broad range of social concerns. In the *COVID-19* treatment, the *WHO Covid-19 Fund* was added to the list of charitable organizations used in *Baseline*, leading to a total of nine charities. In *Covid-19 Only*, the *WHO Covid-19 Fund* was the only available recipient^4^. In all treatments the decision screen included the mission statement of each of the charities. In the *Baseline* and *COVID-19* treatments, participants could distribute their endowment across multiple charities, if any, and themselves. In all treatments, donations were matched at a rate of 25%, i.e., we donated an additional 25% to all donations made by participants. This mechanism ensures that is is socially efficient for the participants to make donations via the donation task that we offer, as opposed to keeping the full endowment themselves and making donations to their preferred charities outside of the experiment. The individual earnings of the experiment are defined by the amount (of the €3 endowment) that subjects kept for themselves. The instructions of the experiment are presented in section A of the Supplementary Material^5^.

After completing the donation task, subjects answered a questionnaire containing subjects' socio-demographic characteristics; subjects' perception of how relevant a charity's work is regarding alleviating the consequences of the Covid-19 pandemic, the perception of the national vs. international assistance offered by the charity, and their trustworthiness; participants' risk perceptions, actions, and motives regarding the Covid-19 pandemic, the climate crisis, and poverty, respectively; as well as subjects' history of donation and work for charities (see section B of the Supplementary Material for the detailed survey questions). Survey items on risk perceptions, actions, and motives are *z*-standardized (across all three treatments in the main experiment). The measures used in the analyses are constructed as the sum of the standardized responses of the items belonging to the particular inventory; this measure is finally *z*-standardized again, such that all measures used in the analyses have a mean of zero and a standard deviation of one. Likewise, survey responses on participants' trust in the charities, their perceived relevance of the charities' work during the pandemic, and the perceived help of the charities during the pandemic are *z*-standardized.

###  Experimental Procedures

A total of 1,762 subjects (*Baseline*: *n* = 581; *COVID-19*: *n* = 599; and *Covid-19 Only*: *n* = 582) were recruited from the standard student subject pool of the University of Innsbruck, Tyrol (Austria) using *hroot* (Bock et al., 2014). As part of western Austria, Tyrol was among the regions that were worst affected by the first wave of the Covid-19 pandemic in Austria, bordering the North of Italy and the South of Germany. The region reported the first cases on February 25, 2020 and entered a lock-down of all municipalities in the region for about 7 weeks on March 16, 2020. During the period of the data collection (April 2020–January 2021), the number of cases in Tyrol varied substantially. During this period, the 7-day incidence varied between 771.3 and 0.1, with peaking values during the months of November and December 2020 and lowest values for May and June 2020 (https://bit.ly/2SOSsFM). Thus, the study covers a period of time with substantial variance in terms of the severity of the Covid-19 pandemic.

We ran the experiments online. Subjects only participated in one of the treatment conditions in a between-subjects design and could only participate once. For each date at which data was collected, invitations were made for three simultaneously running sessions, one for each treatment condition, with up to 40 participants in each treatment, leading to a total number of 16 sessions. Upon receiving the invitation, subjects were informed that this was an online experiment that would last approx. 20 min. As payment options we offered transactions via PayPal or in the form of Amazon gift cards.

We collected data in two different intervals. First, in April and May 2020, we collected weekly data on 1 day of each week, for a total of eight consecutive weeks. Thereafter, starting in June 2020, we collected monthly data on 1 day of each month for a total of eight consecutive months. Subjects were told that they could participate in the experiment as soon as they received the link which was distributed at 10 a.m., and that participation was possible until 8 p.m. on the same day; at 8 p.m., the experimental sessions would be closed and the links would be deactivated.

At the end of each experimental session, the sum of donations across all treatments was transferred to each of the organizations via bank transfers, including a matching payment of 25%. A depersonalized summary of all individual donations as well as the total amount of money paid to each organization was made available on the website of the corresponding author after each experimental session. The payment to participants was transferred within three working days by one of the co-authors.

## 4. Results

The presentation of results is organized in two subsections. First, we focus on average treatment effects. We observe that introducing the *WHO* Covid-19 Solidarity Response fund significantly reduces the average sum of donations to the original eight charities. When looking at the evolution of donations over time, we do do not observe general systematic trends in donations to the different treatment conditions.

Next, we analyze the determinants of individual donations using the data from the post-experiment questionnaire as well as epidemiological data. Our main results show that systematic predictors of total donations are the epidemiological situation, gender, previous charity donations, as well as self-reported Covid-19 actions, poverty risk perceptions and actions. Further, while the 7-day incidence rates and the self-reported Covid-19 risk perceptions do correlate, the epidemiological situation does not significantly explain donations to the *WHO Covid-19 Fund*.

In the following, we will distinguish between (i) the average total donations (*avg. total*) pooled across all charities available as recipients in the respective treatment; (ii) the average sum of donations to the original eight charities in *Baseline* (and thus a subset of the charities in *COVID-19*, excluding the *WHO Covid-19 Fund*; *avg. sum-8*); and finally (iii) the donations to the *WHO Covid-19 Fund* in *COVID-19* or *Covid-19 Only* (*avg. WHO donations*).

###  Treatment Effects

**Figure 1:**
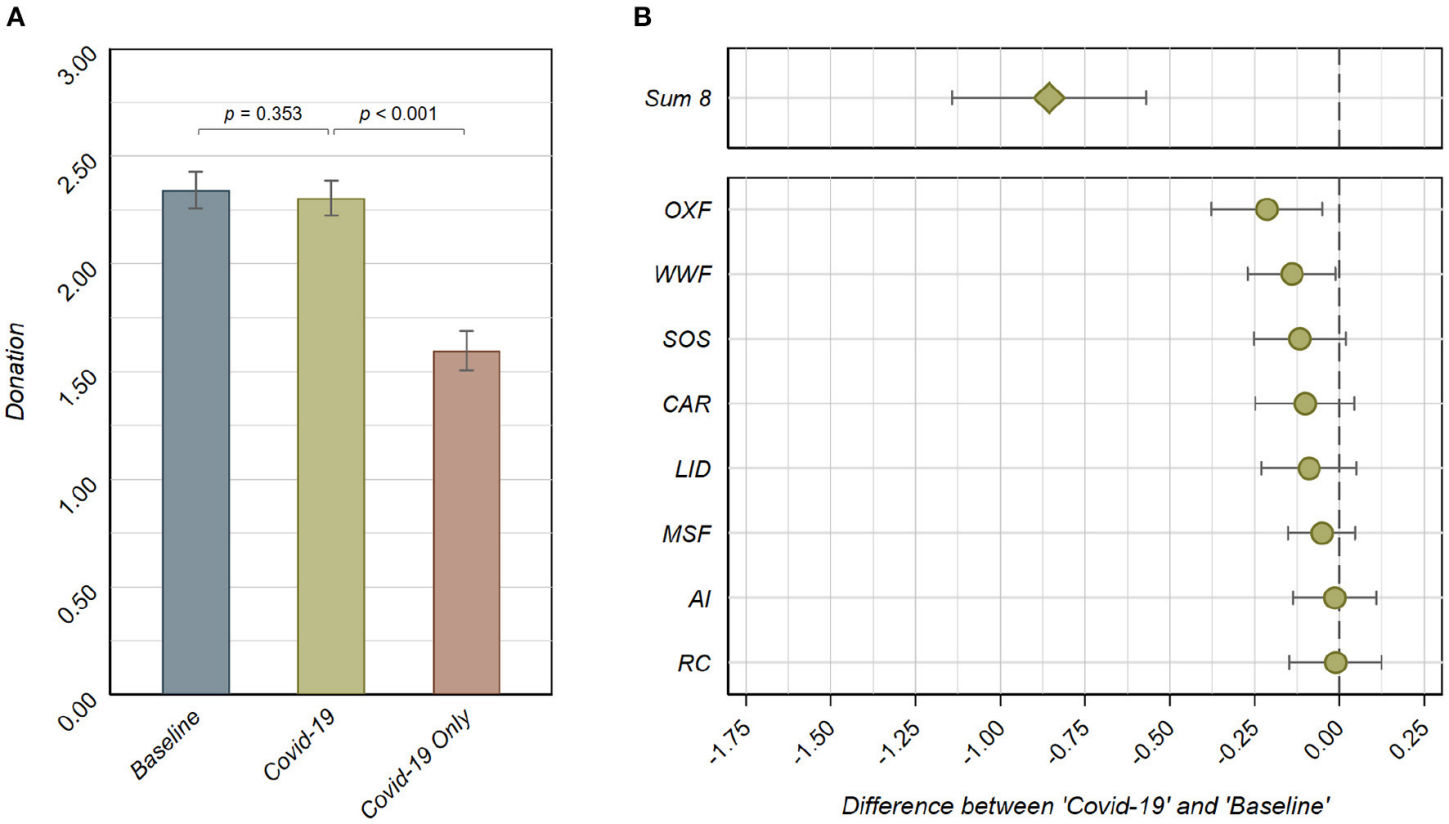
**(A)** *avg. total* donations (pooled across charities) per treatment in €. *p*-values are based on Tobit regressions with €0 and €3 as the lower and upper limit, respectively (endowment €3), and robust standard errors. **(B)** Point estimates and 95% confidence intervals of the differences in *avg. sum-8* donations between the *Baseline* and the *COVID-19* treatment, based on Tobit regressions of the amount donated to the respective charitable organization on a treatment indicator for the *COVID-19* treatment (with €0 and €3 as the lower and upper limit, respectively, and robust standard errors). Negative values represent lower donations in the *COVID-19* treatment than the *Baseline* treatment. All pairwise comparisons between coefficients based on Wald tests after seemingly unrelated regressions (with robust standard errors) are insignificant, except for *OXF*–*AI* (χ^2^(1) = 3.949, *p* = 0.047). The estimate at the top indicates the difference in the sum of donations to the eight charitable organizations between the *Baseline* and the *COVID-19* treatment (*t*(1178) = 5.851, *p* < 0.001; *n* = 1, 180).

When considering the pooled donation data from April 2020 to January 2021, we observe a substitution of social concerns under the presence of the *WHO Covid-19 Fund*. In the *COVID-19* treatment, the average donation to the eight charities is 68.8% of the endowment (*m* = €2.06, *sd* = €1.02), which is significantly lower than the average donation of 78.1% of the endowment in *Baseline* [*m* = €2.34, *sd* = €1.04; *t*_(1,178)_ = 5.851, *p* < 0.001, *n* = 1, 180; see Figure 1B]. Moreover, a Komogorov-Smirnov for the equality of the distribution functions of *avg. sum-8* between *Baseline* and *COVID-19* indicates that the distributions of donations to the eight charities differ systematically between the two treatments (*D* = 0.236, *p* < 0.001; *n* = 1, 180).

This substitution is the sum of consistent but small substitutions for each individual charity. The *avg. sum-8* is smaller in *COVID-19* than in *Baseline* for all charities (negative estimates in Figure 1B), despite these differences being only statistically significant for *OXF* [*t*_(1,178)_ = 2.559, *p* = 0.011; *n* = 1, 180] and *WWF* [*t*_(1,178)_ = 2.119, *p* = 0.034; *n* = 1, 180]. Moreover, all charities are similarly affected by the presence of the *WHO Covid-19 Fund*. In particular, we do not observe significant differences in substitution effects between the different charities, with the only exception being a marginally stronger reduction in donations to *Oxfam* as compared to the reduction in donations to *Amnesty International* [χ^2^(1) = 3.949, *p* = 0.047].

The differences in *avg. sum-8* between *COVID-19* and *Baseline* are not due to a change in the share of participants giving to any of the eight organizations: Pooled across the eight charities, 89.50% and 89.48% of participants choose to donate a positive amount of their endowment in *Baseline* and *COVID-19*, respectively [Pearson's χ^2^-test: χ^2^(1) < 0.001, *p* = 0.992]. The average differences result from the fact that those who donate to any of the eight charities, indeed donate significantly lower amounts in *COVID-19* (*m* = 2.31, *sd* = 0.69) as compared to *Baseline* [*m* = 2.62, *sd* = 0.69; *t*_(1,054)_ = 7.982, *p* < 0.001]. On the charity level, the proportion of participants giving any positive amount, jointly with the amount given by those who donate, separated by treatments are shown in Table 1. While the share of participants donating to charity vary substantially between charities, differences between treatments are not significant, except for the *proportion* of participants donating to *OXF*, which is—as compared to *Baseline*—significantly smaller in *COVID-19* [Pearson's χ^2^-test: χ^2^(1) = 4.793, *p* = 0.029]. Similarly, the *average amount* donated (by those participants who give a positive amount) does not significantly differ between treatments *Baseline* and *COVID-19* for any of the charities, except for the comparison regarding donations to *WWF* [*t*_(583)_ = 2.246, *p* = 0.025].

**Table 1:** Share of participants donating any positive amount, and average amounts donated by those who donate, separated by charities and treatments.

	**Share of donors**				**Avg. Amount donated**			
**Charity**	**Baseline (%)**	**COVID-19 (%)**	**χ^2^(1)**	**p-value**	**Baseline (%)**	**COVID-19 (%)**	**t-value**	**p-value**
*WWF*	51.5	47.8	1.630	0.202	0.90	0.80	2.246	0.025
*MSF*	65.6	62.4	1.261	0.261	0.88	0.87	0.132	0.895
*SOS*	35.1	31.6	1.682	0.195	0.72	0.65	1.452	0.147
*AI*	44.1	44.4	0.014	0.905	0.79	0.75	0.932	0.352
*CAR*	24.1	20.0	2.834	0.092	0.54	0.59	0.852	0.395
*LID*	27.5	25.2	0.825	0.364	0.63	0.56	1.291	0.198
*OXF*	21.3	16.4	4.793	0.029	0.60	0.49	1.898	0.059
*RC*	36.1	36.7	0.043	0.835	0.76	0.71	1.050	0.295

*χ^2^ statistics and the corresponding p-values are reported for treatment comparisons of the share of participants; for comparisons of the average amount donated by those who donate between treatments, the t-statistics and p-values are obtained from Tobit regressions of the amount donated to the respective charitable organization on a treatment indicator for the COVID-19 treatment (with €0 and €3 as the lower and upper limit, respectively, and robust standard errors)*.

The main result on the substitution of social concerns related to the presence of the *WHO Covid-19 Fund* derives from two observations. First, the *avg. WHO donations* are substantial (*Observation 1*). Second, *avg. total* donations do not significantly differ between *Baseline* and *COVID-19*, introducing the *WHO Covid-19 Fund* (*Observation 2*).

The first observation is based on the finding that in the *COVID-19* treatment, with the list of nine charities, donations to *WHO Covid-19 Fund* amount to 8.0% of the endowment (*m* = €0.24, *sd* = €0.48). In particular, the donations to *WHO Covid-19 Fund* significantly exceed the donations to three out of the eight charities (*CAR, LID*, and *OXF*); for two more charities, donations do not significantly differ from donations to the *WHO Covid-19 Fund* (*SOS*, and *RC*). Moreover, when participants can only decide between donating to a Covid-19 charitable organization or keeping money for themselves (*Covid-19 Only*), donations to the *WHO Covid-19 Fund* amount to 53.3% of the endowment (*m* = €1.60, *sd* = €1.12; see Figure 1A).

With respect to the second observation, we do not find evidence for differences in *avg. total* donations between *Baseline* and *COVID-19*. The average total donations in *Baseline* are 78.1% of the endowment (*m* = €2.34, *sd* = €1.04; see Figure 1A). The aggregate donations to the full set of nine charities in the *COVID-19* treatment is slightly lower, at 76.9% of the endowment (*m* = €2.31, *sd* = €1.02), with the difference not being statistically significant [*t*_(1,178)_ = 0.930, *p* = 0.353, *n* = 1, 180; see Figure 1A]. Thus, despite having more possible recipients, the donations do not increase in the *COVID-19* treatment.

**Figure 2:**
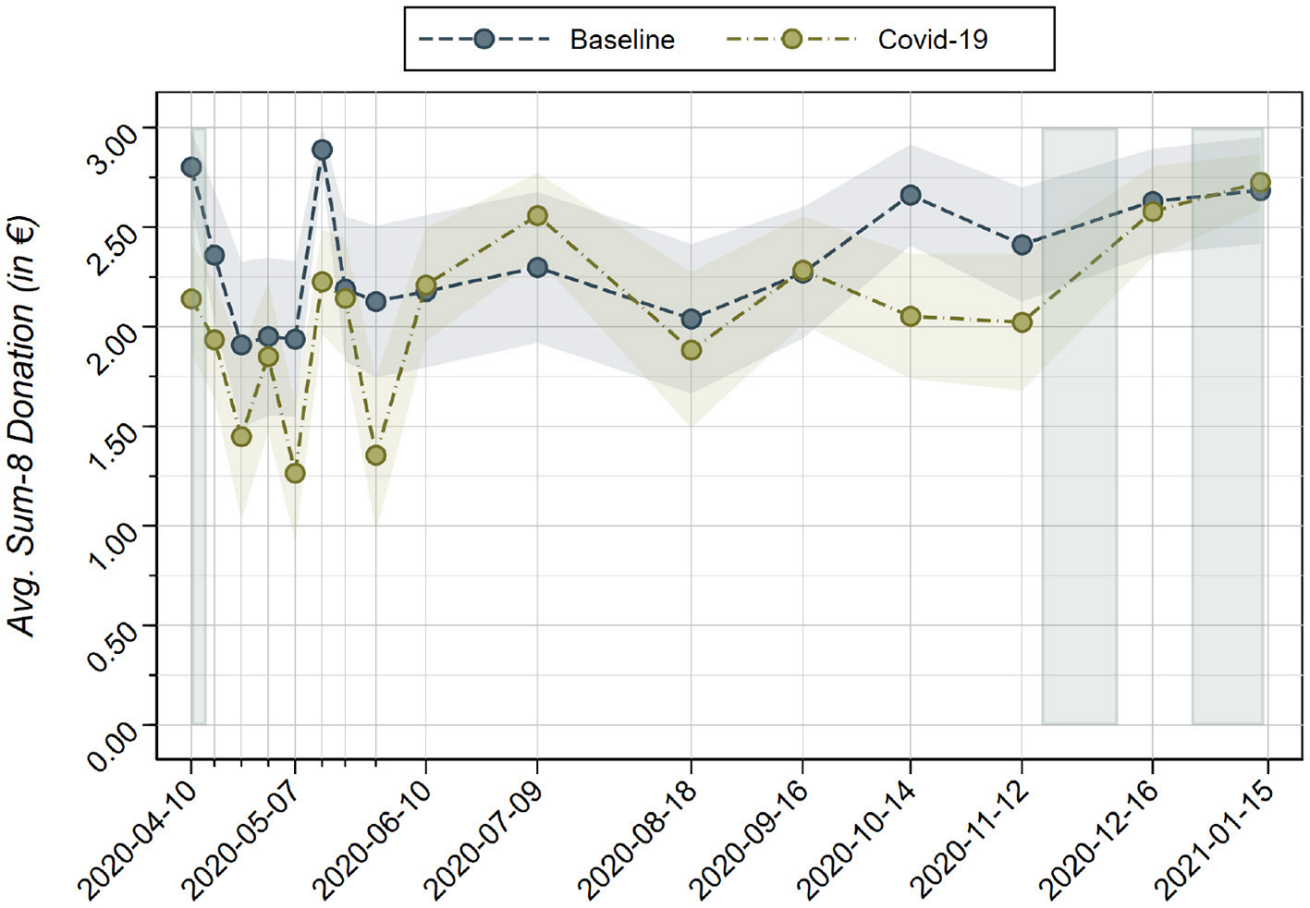
Evolution of *avg. sum-8* donations (in €) in *Baseline* and *COVID-19* per treatment over the eight consecutive weeks plus eight consecutive months of data collection. Shaded areas indicate 95% confidence intervals. Vertically shaded areas indicate lockdown periods. The differences (based on Tobit regressions of *avg. sum-8* on a treatment indicator, with €0 and €3 as the lower and upper limit, respectively, and robust standard errors) between treatments *Baseline* and *COVID-19* are insignificant for each date, except for 2020-04-10 [*t*_(75)_ = 4.094, *p* < 0.001], 2020-04-16 [*t*_(75)_ = 2.202, *p* = 0.031], 2020-05-07 [*t*_(69)_ = 2.501, *p* = 0.015], 2020-05-14 [*t*_(83)_ = 3.805, *p* < 0.001], 2020-05-28 [*t*_(68)_ = 2.644, *p* = 0.010], and 2020-10-14 [*t*_(73)_ = 3.343, *p* = 0.001].

Figure 2 displays the evolution of the *avg. sum-8* donations in *Baseline* and *COVID-19* over time. Looking at the figure we do not observe a systematic time trend in either of the two treatments. Despite the extended time under consideration (10 months) and the convulsive social situation during this period, the donation to the initial list of eight charities fluctuates up to 30 percent of the value without a clear time trend. Second, we do not observe a clear time trend in treatment effects. We observe that in five out of the first 8 weeks (April to May 2020) the *avg. sum-8* donations in *Baseline* are significantly above those in *COVID-19*. Over the summer of 2020, the difference in donations to the original eight charities in *Baseline* and *COVID-19* disappears, and returns only in October, the month that led to the beginning of the *second wave* in Austria, at a level that is comparable to that of early April 2020. Finally, in December and January the difference vanishes again. These results are consistent with the substitution being stronger when the epidemiological situation worsen. But these estimates need to be taken very carefully, as they are based on reduced sub-samples for each time period (roughly 40 observations per treatment each).

Supplementary Figures S2, S3 show the time evolution with respect to *Observation 1* on *avg. WHO donations* and *Observation 2* on *avg. total*, respectively. Generally, for both observations we do not find evidence for systematic variation over time. Figure 2 shows that over time the *avg. WHO donations* in *COVID-19* and *Covid-19 Only* remain above zero throughout all 10 months in both treatments. When the *WHO Covid-19 Fund* is the only possible recipient (*Covid-19 Only*), we observe high variability during the first weeks of Spring 2020 followed by a mild increasing trend after August 2020. When the *WHO Covid-19 Fund* is one of the possible alternative recipients, we do not observe such an evolution. Indeed, the Spearman correlation between donations to the *WHO Covid-19 Fund* in treatments *COVID-19* and *Covid-19 Only* over time turns out to be close to zero (ρ_*s*_ = 0.021, *p* = 0.940; *n* = 16). Finally, we observe that in October 2020 and November 2020, right before the second lock down in Austria, donations to *WHO Covid-19 Fund* in *COVID-19* are lowest; participants seem to prioritize other social concerns at that time.

**Figure 3:**
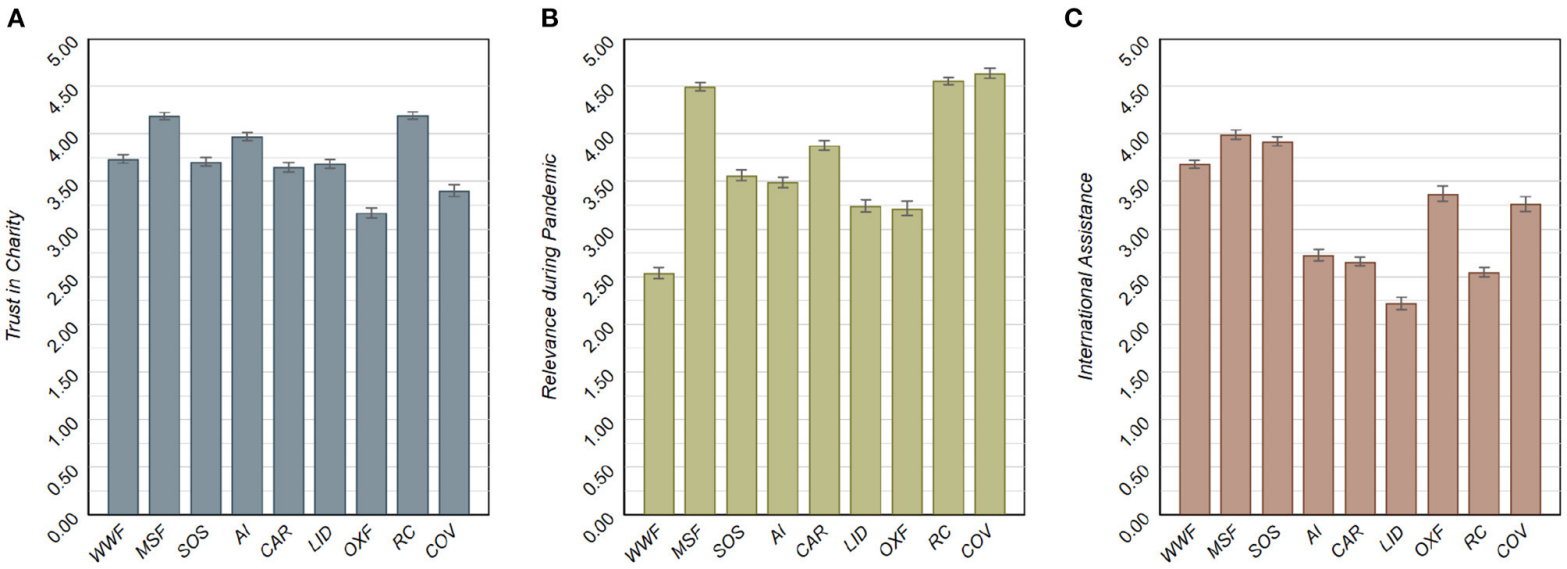
Mean (unstandardized) survey responses on **(A)** trust on the charity, **(B)** relevance of the charity work in fighting the Covid-19 pandemic, and **(C)** the extent to which the work of the charity is perceived to be internationally oriented, separated by the eight charities and the *WHO Covid-19 Fund*. Error bars indicate 95% confidence intervals.

Further, Figure 3 shows the variation in *avg. total* donations over time in all three treatment conditions, as related to *Observation 2*. Remarkably, *avg. total* donations in *Baseline* do not significantly differ from *COVID-19*, except for May 14, 2020 [*t*_(83)_ = 2.192, *p* = 0.031] and October 2020 [*t*_(73)_ = 2.909, *p* = 0.005]. Aggregate pro-social concerns seem to be consistently unaffected by the presence or absence of the *WHO Covid-19 Fund* in the list of recipients throughout the 10 months of the study.

###  Determinants of Individual Donations

In this section, we use participants' responses to the questionnaire and epidemiological data to explore their relevance for donation behavior. On average, participants in our sample are 23.4 years old, and 58.3% of our participants are female; 44.2, 33.8, and 18.4% are of the Austrian, German, and Italian nationality, respectively. 36.6% of our sample has indicated to have donated to a charitable donation in the past 12 months (in reference to the day of participation) and 23.3% have indicated to have volunteered for a charitable organization in the past 12 months.

For each of the charities available as a potential recipient in the donation experiment, Figure 3 presents the mean (unstandardized) survey responses on (a) trust in the charity's work, (b) its perceived relevance in fighting the consequences of the pandemic, as well as (c) the perceived level of international assistance. Generally, we observe relatively high and similar average trust levels for each of the nine charities. There are substantial differences in the perceived relevance between the charities in fighting the consequences of the Covid pandemic, with *MSF, RC*, and the *WHO Covid-19 Fund* showing equally high average levels, and *WWF* having the lowest score. Finally, we observe that the help of *AI, CAR, LID*, and *RC* is perceived to be more nationally oriented than that of the other charities under consideration.

Table 2 presents regression analyses for *avg. total* donations for the pooled data. Model 1 examines the impact of the 7-day incidence rate (in logs) of Covid-19 infections in Tyrol; model 2 additionally includes subjects' socio-demographic characteristic, whether they are a member of a charity, as well as their history of charitable work and donations to charities. Model 3 also incorporates participants' self-reported risk perceptions, actions, and motives related to the Covid-19 pandemic, and finally model 4 incorporates also the risk perceptions, actions, and motives related to climate change and to poverty. Looking at the epidemiological situation and Covid-19 perceptions, we observe a significant correlation between the 7-day incidence and the standardized responses to Covid-19 risks (Pearson correlation: ρ = −0.097, *p* < 0.001), Covid-19 actions (Pearson correlation: ρ = −0.188, *p* < 0.001), and Covid-19 motives (Pearson correlation: ρ = 0.050, *p* = 0.035). While the 7-day incidence rate is an objective measure of the epidemiological situation, the Covid-19 risks, actions, and motives give a subjective measure of the understanding of the pandemic and the epidemiological situation.

**Table 2:** Regression analyses of total donations (pooled across all charities and all treatments) on 7-day incidence rates and individual-level characteristics.

	**(1)**	**(2)**	**(3)**	**(4)**	**(5)**
7-day incidence (log)	0.126^***^	0.123^***^	0.125^***^	0.138^***^	0.132^***^
	(0.029)	(0.028)	(0.028)	(0.029)	(0.028)
Age (in Years)		–0.024	–0.028	–0.017	-0.017
		(0.017)	(0.017)	(0.017)	(0.018)
Female		1.020^***^	1.007^***^	0.855^***^	0.705^***^
		(0.137)	(0.136)	(0.135)	(0.134)
Germany		–0.126	-0.102	–0.066	–0.131
		(0.149)	(0.148)	(0.147)	(0.147)
Italy		–0.317	–0.278	–0.239	–0.223
		(0.179)	(0.180)	(0.180)	(0.177)
Other Country		0.204	0.180	0.199	0.179
		(0.368)	(0.366)	(0.354)	(0.355)
Charity member			–0.037	–0.059	–0.099
			(0.176)	(0.175)	(0.174)
Charity Work			0.029	0.017	–0.089
			(0.164)	(0.162)	(0.163)
Charity Donations			0.451^**^	0.404^**^	0.232
			(0.140)	(0.138)	(0.141)
Covid-19: Risks				–0.091	–0.128
				(0.078)	(0.078)
Covid-19: Actions				0.278^***^	0.258^**^
				(0.078)	(0.079)
Covid-19: Motives				0.217^**^	0.124
				(0.084)	(0.092)
Climate: Risks					0.135
					(0.089)
Climate: Actions					0.123
					(0.088)
Climate: Motives					–0.021
					(0.112)
Poverty: Risks					–0.158^*^
					(0.076)
Poverty: Actions					0.272^**^
					(0.089)
Poverty: Motives					0.096
					(0.098)
Constant	2.564^***^	2.627^***^	2.538^***^	2.324^***^	2.544^***^
	(0.102)	(0.445)	(0.443)	(0.443)	(0.457)
Observations	1,762	1,751	1,751	1,751	1,751
Pseudo *R*^2^	0.004	0.018	0.020	0.028	0.035

*The table reports the results of Tobit regressions with €0 and €3 as the lower and upper limit, respectively. Robust standard errors are provided in parentheses*.

* *p < 0.05*,

** *p < 0.01*,

*** *p < 0.001*.

First, we observe that the 7-day incidence as a measure for the epidemiological situation is a significant determinant of individuals' donation behavior in all model specifications. Furthermore, we report that females donate significantly more and subjects having donated to charities in the past are also associated with significantly higher total donations in the donation task. Finally, we do not find evidence that self-reported Covid-19 risk perceptions are a significant predictor of donations, but we observe that Covid-19 actions and motives show a significant and positive relationship with donations. The Covid-19 motives are however not significant after controlling for the additional variables in Model 5. Perceptions of risks associated with poverty are negatively correlated with donations, while poverty actions have a positive and significant impact. We do not observe significant effects of perceived risks, motives, or actions related to climate change on total donations.

Table 3 presents the model in Model 5 of Table 2, including in addition the charity-specific self-reported degree of trust on the charity, the perceived relevance of the charity in fighting the consequences of the Covid-19 pandemic, and whether the charity is perceived to provide assistance internationally or nationally. The results show that the trust in the charity have a significant positive effect on donations for each individual charity. Interestingly, the epidemiological data does not significantly correlate with donations to any of the different charities after controlling for subjects' perception about each charity. The rest of the variables significantly affect donations for some of the charities, but not generally for all of them.

**Table 3:** Regression analyses of donations to charity (pooled across all treatments) on 7-day incidence rates and individual-level characteristics, separated by the eight charities and the *WHO Covid-19 Fund*.

	***WWF***	***MSF***	***SOS***	***AI***	***CAR***	***LID***	***OXF***	***RK***	***COV***
7–day incidence (log)	0.009	0.019	0.013	0.023	–0.009	0.002	–0.013	–0.040^*^	0.011
	(0.014)	(0.012)	(0.016)	(0.016)	(0.019)	(0.016)	(0.024)	(0.017)	(0.044)
Age (in Years)	0.018	0.007	–0.025^*^	0.007	–0.028^*^	–0.006	–0.010	–0.016	0.048
	(0.012)	(0.010)	(0.012)	(0.012)	(0.013)	(0.010)	(0.016)	(0.012)	(0.038)
Female	0.152	0.180^*^	0.169	0.103	0.089	0.325^***^	0.383^**^	0.252^**^	0.231
	(0.083)	(0.070)	(0.095)	(0.084)	(0.106)	(0.090)	(0.128)	(0.094)	(0.256)
Germany	0.076	0.102	0.105	0.088	–0.058	0.287^**^	–0.187	–0.247^*^	–0.125
	(0.083)	(0.073)	(0.098)	(0.093)	(0.113)	(0.107)	(0.131)	(0.101)	(0.265)
Italy	0.109	0.214^*^	–0.218	–0.000	–0.241	0.080	–0.273	0.057	–0.451
	(0.110)	(0.083)	(0.129)	(0.117)	(0.139)	(0.114)	(0.197)	(0.119)	(0.340)
Other Country	0.303	0.254	0.465^*^	0.154	0.495^**^	0.282	0.267	0.183	–1.304^*^
	(0.178)	(0.136)	(0.192)	(0.180)	(0.190)	(0.196)	(0.270)	(0.175)	(0.582)
Charity Member	–0.005	0.038	–0.127	0.082	0.077	0.196	–0.133	0.269^*^	0.002
	(0.104)	(0.084)	(0.116)	(0.100)	(0.125)	(0.110)	(0.155)	(0.126)	(0.296)
Charity Work	0.017	0.042	0.168	–0.041	–0.065	–0.121	0.146	0.100	0.128
	(0.092)	(0.078)	(0.107)	(0.103)	(0.108)	(0.099)	(0.143)	(0.110)	(0.285)
Charity Donations	0.110	–0.097	–0.030	–0.160	0.079	–0.020	0.127	–0.027	–0.216
	(0.079)	(0.067)	(0.088)	(0.087)	(0.107)	(0.092)	(0.123)	(0.097)	(0.245)
Covid–19: Risks	–0.020	–0.022	0.070	0.047	–0.054	0.035	–0.018	0.096^*^	0.084
	(0.044)	(0.038)	(0.050)	(0.046)	(0.061)	(0.045)	(0.067)	(0.046)	(0.146)
Covid–19: Actions	–0.067	0.044	–0.062	0.007	–0.151^**^	0.001	–0.153	0.001	0.024
	(0.050)	(0.039)	(0.054)	(0.052)	(0.059)	(0.051)	(0.080)	(0.055)	(0.143)
Covid–19: Motives	–0.036	–0.001	–0.069	–0.085	0.107	0.016	–0.027	–0.050	0.040
	(0.050)	(0.045)	(0.065)	(0.056)	(0.067)	(0.057)	(0.091)	(0.060)	(0.151)
Climate: Risks	0.141^**^	–0.054	–0.033	–0.088	–0.024	–0.009	–0.081	–0.040	–0.034
	(0.052)	(0.041)	(0.058)	(0.058)	(0.065)	(0.053)	(0.080)	(0.055)	(0.162)
Climate: Actions	0.076	–0.010	–0.013	–0.043	–0.004	–0.003	–0.037	–0.021	0.025
	(0.052)	(0.038)	(0.055)	(0.047)	(0.065)	(0.053)	(0.066)	(0.056)	(0.163)
Climate: Motives	0.161^**^	–0.015	–0.076	–0.004	–0.181^*^	–0.192^**^	0.147	–0.118	–0.385
	(0.058)	(0.050)	(0.074)	(0.066)	(0.078)	(0.059)	(0.090)	(0.067)	(0.220)
Poverty: Risks	0.004	–0.104^**^	–0.061	–0.076	–0.044	–0.016	0.058	–0.051	0.039
	(0.045)	(0.038)	(0.054)	(0.047)	(0.054)	(0.046)	(0.068)	(0.049)	(0.140)
Poverty: Actions	–0.093	0.085^*^	0.238^***^	–0.049	0.075	0.057	0.137	0.038	0.142
	(0.050)	(0.040)	(0.062)	(0.057)	(0.061)	(0.054)	(0.086)	(0.060)	(0.171)
Poverty: Motives	–0.109	0.013	–0.042	0.080	0.058	0.057	–0.207^*^	0.051	0.201
	(0.056)	(0.055)	(0.070)	(0.059)	(0.068)	(0.060)	(0.097)	(0.074)	(0.188)
Trust in Charity	0.352^***^	0.311^***^	0.374^***^	0.372^***^	0.376^***^	0.294^***^	0.362^***^	0.414^***^	0.545^***^
	(0.043)	(0.034)	(0.056)	(0.044)	(0.054)	(0.042)	(0.060)	(0.051)	(0.133)
Relevance during Pandemic	0.083^*^	0.036	0.103^*^	0.070	0.067	0.060	0.144	0.034	0.118
	(0.039)	(0.030)	(0.048)	(0.042)	(0.046)	(0.041)	(0.074)	(0.051)	(0.125)
International Assistance	–0.005	0.108^***^	–0.028	0.011	0.111^*^	–0.160^***^	0.164^*^	0.003	–0.067
	(0.036)	(0.032)	(0.040)	(0.042)	(0.047)	(0.041)	(0.063)	(0.041)	(0.119)
Constant	–0.535	–0.013	0.142	–0.349	–0.116	–0.498	–0.437	–0.011	–0.712
	(0.295)	(0.252)	(0.298)	(0.307)	(0.329)	(0.274)	(0.412)	(0.294)	(0.952)
Observations	656	685	578	651	656	489	238	696	469
Pseudo *R*^2^	0.110	0.092	0.111	0.073	0.111	0.123	0.227	0.098	0.025

*The table reports the results of Tobit regressions with €0 and €3 as the lower and upper limit, respectively. Robust standard errors are provided in parentheses*.

* *p < 0.05*,

** *p < 0.01*,

*** *p < 0.001*.

## 5. Discussion and Conclusion

This paper presents evidence on long-term (10 months) substitution effects that the Covid-19 pandemic has on other social concerns. We report results from a large online experiment with 1,762 students making real-life donations to charities between April 2020 and January 2021. As apprehended by policy makers, our findings suggest a substitution effect due to the Covid-19 pandemic. The data shows that introducing the *Covid-19 Solidarity Response Fund for WHO* as a potential recipient significantly reduces the donations to the rest of eight organizations, as compared to another treatment where only eight charities comprising a wide range of social concerns are available. This result is driven by two main observations: (i) Participants donate substantial amounts to the *WHO Covid-19 Fund*; and (ii) the total donations are not significantly different when the *WHO Covid-19 Fund* is present. That is, aggregate pro-social concerns do not differ depending on whether the *WHO Covid-19 Fund* is available in the list of charitable organizations participants could donate to. This is in line with previous results for treatment effects reported in Blanco et al. (2020) for the onset of the pandemic.

It is worth emphasizing that these results differ from the results that could be expected to derive from the methodological variation of the number of recipients. Our experimental design implies that there are eight possible recipients in *Baseline*, whereas the number of possible recipients is increased to nine in *COVID-19*. In principle, this variation in the number of possible recipients could already affect the results, rather than (or in addition to) the fact that the *WHO Covid-19 Fund* is the introduced charity. The previous evidence on charity competition reviewed in section 2 suggests that the experimental design would induce higher total donations in *COVID-19* (with nine possible recipients) than in *Baseline* (with eight possible recipients). Previous studies experimentally varying the number of charities to which people can donate have consistently observed increased aggregate levels of total donations (Soyer and Hogarth, 2011; Schmitz, 2021). This is not what we observe in our data. Participants' average total donations actually turn out to be *smaller* in the *COVID-19* treatment with nine possible recipients as compared to the *Baseline* treatment with eight possible recipients. While the difference is not statistically significant, our results do not support an increase in total donations due to an increase in the number of possible recipients. Thus, we believe that the results reported here are a lower bound estimate of the substitution effect due to the presence of the *WHO Covid-19 Fund*.

Providing a full characterization of the impact of including the *WHO Covid-19 Fund* in the treatment comparison would require considering ten different treatment conditions: including all nine charities and a sequential exclusion of one single charity in nine additional treatments. One could then compare the strength of the different treatment effects for the exclusion of the *WHO Covid-19 Fund* as compared to the treatment effect from the exclusion of each other charity. Given the limitations with respect to the number of students in the subject pool we could not run these ten treatments for each data point for 10 months. In this study we have prioritized the use of the subject pool to assess the time evolution of donation behavior. Other studies could focus on the methodological question of assessing to which extent there could be substitution effects from restricting the decision setting to other social causes, and if present, the relative size of the substitution for different social concerns.

Looking into the evolution over time, for the 10 months time period covered by our data, we do not find any indication for systematic trends in donations. As compared to the results in Blanco et al. (2020), additional analyses show no change in the total donations from the first 8 weeks to the subsequent 8 months of data in the *Baseline* nor *Covid-19 Only* treatments. We do observe however a significant increase in donations for the *COVID-19* treatment when comparing the first 8 weeks and the subsequent 8 months of data collection. We observe that for the *COVID-19* treatment there is a significant increase in donations to *AI, CAR*,*WWF, LID*, and *OXF*; there is no significant change for *MSF* and *RC*; and there is a significant decrease for the *WHO Covid-19 fund*. For the *Baseline* and the *Covid-19 Only* treatments there are no significant differences in donations to each charity generally, with the only exception of a significant increase of donations to *WWF* for the *Baseline* treatment.

When looking into the determinants of aggregate donations by participants in our study, we see that there is evidence that the worsening of the epidemiological situation, measured by the 7 day incidence, significantly increases total donations. Moreover, as expected, we find a significant gender effect, with women donating significantly higher total amounts than men. Moreover, people taking actions against Covid-19 and against poverty donate significantly more, whereas people fearing risks from poverty donate significantly less. When looking into separate donations to each charity, we find that trusting a given charitable organization is the strongest explanatory factor of why participants donate to the respective charity.

We believe that our results can be informative to policy makers, helping them better understand human behavior during global shocks such as the Covid-19 pandemic. This global health crisis has been attracting the international community's attention to the interrelation between the environment, health, and inequality in human well-being. At the same time, there is a fear that the pandemic dominates both policy and social agendas, at the expense of other social concerns. We present evidence that such substitution of social concerns is only partially present among the participants in our study. While we observe a reduction of concerns for other (non-Covid-19) social objectives, donations to charities in other domains remain at relatively high levels. This behavior seems to be stable during the pandemic; we do not find clear trends over the 10 months of our study. The aforementioned results suggests an optimistic prospect since it represents a backup for the ongoing considerations with respect to other social concerns that public administrations and charities worldwide have been pursuing before and during global crisis such as the Covid-19 pandemic. It is also worth highlighting how the participants in our study are sensitive to the needs of others, increasing total donations in times of higher incidence rates of Covid-19 infections.

The experimental methodology used in this study inevitably is subject to certain limitations. The experimental design of the donation task allows to draw causal inference with respect to the treatment effects we report, but the donation task under analysis is only a proxy of pro-social behavior in the field. Similarly, as common in economic experiments in the laboratory, our participants form a very homogeneous sample of students with similar age, education level and socio-demographic background. An additional limitation of our study is that the nature of some of our treatments, i.e. *Covid-19* and *Covid-19 Only*, might be subject to some experimenter demand effects (Zizzo, 2010) since the presence of a Covid-19 fund might be very appealing for the participants. However, recent evidence suggests that pro-social behavior in the lab—elicited using a similar donation task—significantly correlates with health behavior during the pandemic in the field (Campos-Mercade et al., 2021). Further, by nature, we cannot analyze the extent to which treatment results would have differed, had we started to collect data prior to the pandemic. The *WHO Covid-19 fund* was established only after the pandemic stroke. Finally, the data used in this project was collected for a pre-determined (and pre-registered) period of 10 months during the pandemic. Certainly, we see value in future research replicating this data collection after this pandemic is over in order to expand our understanding of the very long term effects and the behavior in the aftermath of such unprecedented global shocks.

## Data Availability Statement

The original contributions presented in the study are publicly available. This data can be found here: https://osf.io/uy7ps.

## Ethics Statement

The studies involving human participants were reviewed and approved by Board for Ethical Questions in Science of the University of Innsbruck. The patients/participants provided their written informed consent to participate in this study.

## Author Contributions

EB: conceptualization, experimental design, writing-original draft, supervision, project administration, and funding acquisition. AB: performing experiments and writing-original draft. FH: statistical analysis, data curation, writing-original draft, and visualization of the manuscript. TJ-L: experimental design, statistical analysis, writing-review, and editing. NS: development of the questionnaire, programming, performing experiments, writing-original draft, and project administration. All authors contributed to the article and approved the submitted version.

## Funding

Funding was provided by FWF (No. P 32859).

## Conflict of Interest

The authors declare that the research was conducted in the absence of any commercial or financial relationships that could be construed as a potential conflict of interest.

## Publisher's Note

All claims expressed in this article are solely those of the authors and do not necessarily represent those of their affiliated organizations, or those of the publisher, the editors and the reviewers. Any product that may be evaluated in this article, or claim that may be made by its manufacturer, is not guaranteed or endorsed by the publisher.

## Footnotes

^1^Please see section 3 for a discussion of the methodological implications of changing the number of possible recipient charities and their relevance in the results.

^2^Please note that the current study includes the weekly data from April and May 2020 on which the results in Blanco et al. (2020) are based on, and uses the same treatment variations (see section 3) as the initial study. The subsequent monthly data from June 2020 to January 2021 is reported for the first time in this paper, as are the results related to several of the questionnaire items. Incorporating the relevance of the Covid-19 incidence rate on donation behavior is also a novel aspect of the current study.

^3^A related line of research looking at competition between charities examines the effect of targeting one charity out of a list of potential recipients on donations to other charities. Applied mechanisms are information priming (Harwell et al., 2015; Filiz-Ozbay and Uler, 2019) or different incentives through matching donations (i.e., the experimentalist adds a fix rate to each donation made by participants) (Gallier et al., 2019; Schmitz, 2021). Applying a matching to one charity out of a set does not generate substitution in donations between charities with similar objectives. Schmitz (2021) finds that the matching does not change total net donations to all charities. Gallier et al. (2019) observes increases in net donations to the matched charity and to other similar charities. These studies, however, differ substantially from our design, as we keep constant the incentives to donate to each of the recipient.

^4^Please see section 5 for a discussion of the implications of the methodological aspects of this design on the results. Specifically, addressing how the change in the number of recipients could affect the findings.

^5^Note that in addition to the treatments described here, we conducted a series of robustness sessions, including a 10 fold increase in endowments, with subjects making decisions over €30 in all three decision settings, and additional robustness tests. The results are reported in (Blanco et al., 2020). The observed treatment effects are robust to these changes.
