# Supplementary material for: Long Term Effects of the COVID-19 Pandemic on Social Concerns
Source: Front Psychol. 2021 Oct 5;12:743054. doi: 10.3389/fpsyg.2021.743054 (PMC8525284; doi:10.3389/fpsyg.2021.743054)
Supplement: Supplementary file 1 [file Presentation_1.pdf]

Supplementary Material

Substitution of social concerns under the Covid-19 pandemic

Contents

1. Introduction **2**

2. Related Literature **4**

3. Experimental Design **7**

4. Results **8**

5. Discussion & Conclusion **16**

A Experimental instructions 1

B Survey questions 3

C Supplementary analysis 7

A. Experimental instructions

The instructions were in German. Below (in italics) we present English translations. We present here the instructions used in the *Baseline* and *Covid-19* treatments; for *Covid-19 Only* the wording was adapted such that it corresponds to a donation decision with a single charitable organization (rather than a set of charities). Fig. S1 presents a screenshot from the decision-making screen in *LimeSurvey* for the *Covid-19* treatment.

In this part of the experiment you will be paired with a set of charities. You will be making decisions for a total of 3 Euros; you must decide how many Euros (if any) you allocate to one or several of the charities below and how many Euros (if any) you take for yourself. For every euro you allocate to a charity, we will transfer in addition 25 cents to the charity.

At the end of today's experimental session, the principal investigator of this project, Dr. XXX, will pool the money all participants have allocated to each one of the charities and will make an online payment for the sum of money plus 25 cents for every Euro.

You will have available a list of all individual contributions to each charity (without participant names) as well as the total sum of money paid to each one of the charities on the personal webpage of Dr. XXX.

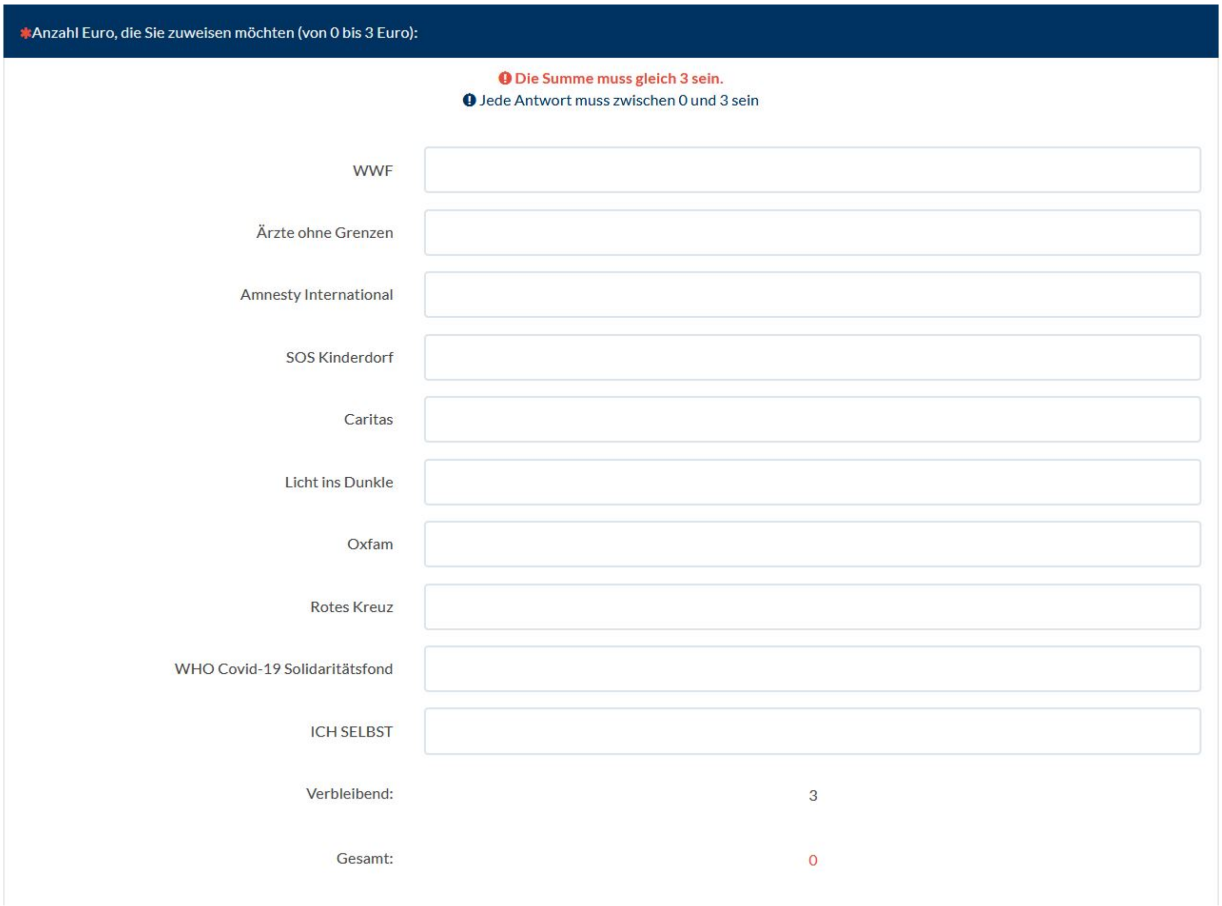

Figure S1: Decision-making screen in LimeSurvey for *Covid-19* treatment

Mission Statements:

WWF's mission is to conserve nature and reduce the most pressing threats to the diversity of life on Earth (https://worldwildlife.org).

Doctors Without Borders provides assistance to populations in distress, to victims of natural or man-made disasters and to victims of armed conflict (https://doctorswithoutborders.org).

Amnesty International is committed to the enforcement of the Universal Declaration of Human Rights and other rights which are part of international human rights agreements (https://amnesty.at).

In the center of **SOS Children's Villages** is the effort to provide children who have lost their parents or can no longer live with them, with a lasting and permanent home and a stable environment (https://sos-kinderdorf.at).

Independent from their social, national or religious affiliation, **Caritas** supports and accompanies people in difficult life situations, who suffer from illness or disability as a consequence of accidents or disasters (https://caritas.at).

The philosophy of the organization **Licht ins Dunkel** is the material and emotional support of disabled children and their families, physically and mentally disabled people in Austria , as well as the promotion of the objectives of its members as set in the organization charta. (https://lichtinsdunkel.orf.at).

Oxfam is an independent relief and development organization. We are convinced that poverty and injustice are preventable and can be overcome (https://oxfam.de).

The Red Cross mission is to improve the lives of people in need and vulnerable groups through the power of humanity (https://roteskreuz.at).

WHO **Covid-19 Solidarity Response Fund:** We are all affected by the growing COVID-19 pandemic. It's an unprecedented health challenge and we know people and organizations everywhere want to help. The World Health Organization is leading and coordinating the global effort, supporting countries to prevent, detect, and respond to the pandemic. (https://covid19responsefund.org).

B. Survey questions

The survey consisted of three main blocks, addressing participants' (a) perception of risks, (b) actions, and (c) motivations related to (i) the Covid-19 pandemic, (ii) the climate crisis, and (iii) poverty. The survey questions used in each of these inventories are provided below. Additionally, participants were asked to answer some general questions on socio-economic details, subjects' perception of how relevant a charity's work is regarding alleviating the consequences of the Covid-19 pandemic, their perceived trustworthiness, as well as subjects' history of donation and charity work. The survey items were displayed to the participants via *LimeSurvey* using matrix questions (or open text field questions, where applicable). We report below the complete ex-post questionnaire. All questions except Q9, Q10, Q18 and Q19 were part of the analyses reported in this study.

Questions on the Covid-19 pandemic

- **Risk perception: (Q1)** To what extent do you agree with the following statements about the Covid-19 disease? (Answers ranging from 1: "I fully disagree" ... to ... 5: "I fully agree"): *(i)* Covid-19 will limit our lives for months; *(ii)* The disease is worse than a conventional flu; *(iii)* The risk of becoming infected with the virus for me or my family and friends is high; *(iv)* I feel anxious when acquaintances are (were) infected with Covid-19; *(v)* I am not concerned with my generation, but with older people in society; *(vi)* A vaccine against the virus will be developed during the summer[6]; *(vii)* There will be (have been) food shortages during the crisis; *(viii)* Due to the restrictions, there will be (has been) a severe economic recession; *(ix)* I am afraid of losing my job because of the situation.

- **Actions: (Q2)** For each of the following actions, please indicate the respective frequency at which you undertake them. (Answers ranging from 1: "Never" ... to ... 5: "Always"): *(i)* I wash my hands at least 20 seconds after having left the house; *(ii)* When I am not at home, I keep a minimum distance of 1.5 meters from other people; *(iii)* I help the group most at risk to do their shopping so that they do not have to leave the house; *(iv)* I am wearing a mask in public; *(v)* I make sure that hygienic measures are also implemented in my house; *(vi)* I try to avoid public transport; *(vii)* I try to go shopping rarely, and only when the shops are less crowded.

  (Q3) How do you rate the following statements in relation to the measures taken in spring 2020 to contain the spread of Covid-19 (e.g., regional lock-downs)? (Answers ranging from 1: "Not true at all" ... to ... 5: "Completely true") *(i)* The measures are (were) annoying me; *(ii)* The measures taken are (were) important and appropriate; *(iii)* The measures taken are (have been) followed by the majority of the population.

- **Motives: (Q4)** To what extend do you agree with the following statements? It is important to me to help contain the spread of Covid-19, because... (Answers ranging from 1: "I fully disagree" ... to ... 5:"I fully agree"): *(i)* I want to protect my fellow human beings; *(ii)* otherwise, I fear that the virus will remain a constant companion; *(iii)* friends or family are (have been) already infected; *(iv)* I think that makes a good citizen; *(v)* I want life to return to normal as soon as possible; *(vi)* I want to follow governmental regulations; *(vii)* a further spread of the virus would threaten me financially; *(viii)* the collapse of the health system must be prevented; *(ix)* the pressure from family

[6] Over the course of our data collection, this question had to be slightly adapted to keep it meaningful. The wording was changed to "A vaccine against the virus will be developed during the next few months" for October, November and December and finally to "The vaccine will enable the end of the pandemic in the next few months " for January.

and friends has a huge impact on me; *(x)* my parents gave me these values; *(xi)* it is important for my religion.

Questions on the climate crisis

- **Risk perception: (Q5)** To what extent do you agree with the following statements? (Answers ranging from 1: "I fully disagree" ... to ... 5: "I fully agree"): *(i)* The risk of severe weather events will increase if society does not act against climate change; *(ii)* Our climate has already changed sustainably; *(iii)* I am afraid of climate change; *(iv)* Climate change will limit our lives; *(v)* Long periods of drought can lead to water shortages also for us; *(vi)* Due to climate change, there will be food shortages; *(vii)* Due to climate change, there will be a severe economic recession; *(viii)* I am afraid of losing my job due to climate change.

- **Actions: (Q6)** For each of the following actions, please indicate the respective frequency at which you undertake them. (Answers ranging from 1: "Never" ... to ... 5: "Always"): *(i)* I use the car; *(ii)* I use a bike or walk; *(iii)* I fly with an airplane; *(iv)* I use public transport; *(v)* I switch my devices to stand-by-mode; *(vi)* I eat meat; *(vii)* I buy seasonal and regional products; *(viii)* I conscientiously separate my waste; *(ix)* I pay a CO2 compensation when buying train, bus or plane tickets.

  (Q7) How would you describe your behavior over the past 12 months on the following points? (Answers ranging from 1: "Reduced by a lot" ... to ... 5: "Increased a lot"): *(i)* The use of cars; *(ii)* The use of public transports; *(iii)* Flying on airplanes; *(iv)* My consumption of meat; *(v)* The production of waste; *(vi)* The payments of CO2 compensations; *(vii)* My consumption of regional and seasonal products.

  (Q8) To what extent do you agree with the following statements? (Answers ranging from I fully disagree ... to ... I fully agree): *(i)* I try to make my consumption as sustainable as possible; *(ii)* I try to reduce my consumption in order to reduce my carbon footprint; *(iii)* Due to climate change, I changed the destination, the length or my general picture of vacations.

  (Q9) For each of the following measures, please indicate whether you or your family have taken them. (Answer possibilities 1: "Yes", 2: "Unsure", 3: "No"): *(i)* Buying insurance due to the risk of flooding, storm and heat damage; *(ii)* Measures to reduce thermal stress e.g. Fan, air conditioning; *(iii)* Protection against flooding e.g. remove valuable items from the basement, sandbags, water pumps; *(iv)* Protection against increased UV radiation, e.g. by applying sun blockers, wearing long clothes or stay in areas protected from the sun.

  (Q10) Have you or your family invested money in any of the above measures in the past 12 months? (Answers possibilities 1: "No, none of these measures have been taken", 2: "No, the measures that were taken were free", 3: "• Yes, in total approximately this amount:...").

- **Motives: (Q11)** To what extent do you agree with the following statements? It is important to me to reduce my influence on climate change because... (Answers ranging from 1: "I fully disagree" ... to ... 5: "I fully agree"): *(i)* I care about the well-being of future generations; *(ii)* I represent values that rely on sustainability; *(iii)* I care about the planet's biodiversity; *(iv)* I believe that climate protection makes a good citizen; *(v)* it is an important topic in my circle of friends; *(vi)* my parents gave me values that concern sustainability; *(vii)* it is important for my religion; *(viii)* Climate change may weaken our economy sustainably; *(ix)* I fear that environmental disasters will increase; *(x)* I care about the living conditions in the future; *(xi)* my family is directly affected by climate change; *(xii)* I want to continue planning my vacation regardless of climate change.

Questions on poverty

- **Risk perception: (Q12)** To what extent do you agree with the following statements? (Answers ranging from 1: “I fully disagree” ... to ... 5: “I fully agree”): *(i)* Severe poverty will lead to further refugee crises; *(ii)* Crime is a risk factor that arises from poverty; *(iii)* Global poverty has worsened in the past 12 months; *(iv)* I am afraid that the consequences of poverty will affect me; *(v)* Tourism is negatively impacted by the uncertainty that results from crime in low-income countries.

- **Actions: (Q13)** For each of the following statements, please indicate the respective frequency at which you have undertaken them in the past 12 months. (Answers ranging from 1: “Never” ... to ... 5: “Always”): *(i)* I gave money to poor people; *(ii)* I bought food for poor people; *(iii)* I bought fair produced products; *(iv)* I took vacations that are morally acceptable .

  (Q14) To what extent do you agree with the following statements? (Answers ranging from 1: “I fully disagree” ... to ... 5: “I fully agree”): *(i)* I am worried about the poverty in the world; *(ii)* I am concerned about the poverty in my country; *(iii)* I am concerned about the refugee crisis; *(iv)* I think everyone should pay taxes to have a fair redistribution system; *(v)* I take care of the welfare of refugees.

- **Motives: (Q15)** To what extent do you agree with the following statements? It is important to me to participate in the fight against poverty because... (Answers ranging from 1: “I fully disagree” ... to ... 5: “I fully agree”): *(i)* I care about the well-being of future generations; *(ii)* My values stand for a fair world; *(iii)* I am aware that I was privileged growing up in a developed country; *(iv)* Many low-income countries will become even poorer without interventions; *(v)* I believe that participating in the fight against poverty makes a good citizen; *(vi)* My parents gave me values that encourage me to act against poverty; *(vii)* It is important for my religion; *(viii)* I am afraid that poverty will increase crime that can affect me; *(ix)* Poverty and refugee waves could weaken our economy sustainably; *(x)* I fear that otherwise there will be more refugee crises; *(xi)* I do not want to make my future vacation plans dependent on poverty problems in other countries.

Additional Questions

- **Socio-economic questions: (Q16)** *(i)* What is your gender?; *(ii)* How old are you?; *(iii)* What is your nationality?; *(iv)* What is your marital status?; *(v)* What is your field of study?; *(vi)* In which countries or regions have you been living since February 2020?; *(vi)* In which countries or regions have your family and close friends been living since February 2020?.

- **History of donation and volunteering: (Q17)** *(i)* Are you a member of charity or non-governmental organizations (NGOs)?; *(ii)* Have you volunteered for charitable organization(s) in the past 12 months? ; *(iii)* Have you donated to one or more charities or NGOs during the past year? ;

- **General questions to the organisations and questions on trust: (Q18)** How much do you trust the people in the following groups? (Answers ranging from 1: “Trust completely” ... to ... 5: “Do not trust at all”): *(i)* Your family; *(ii)* Your neighbors; *(iii)* Other students of the University of Innsbruck; *(iv)* Somebody you meet for the first time.

  (Q19) I know the work of the organisation: (Answers ranging from 1: “Very well” ... to ... 5: “Never heard before”): *(i)* WWF; *(ii)* Doctors Without Borders; *(iii)* Amnesty International; *(iv)* SOS Kinderdorf; *(v)* Caritas; *(vi)* Licht ins Dunkel; *(vii)* Oxfam; *(viii)* Red Cross; *(ix)* WHO Covid-19 Response Fund.

(Q20) I trust the work of the organisation: (Answers ranging from 1: “Trust completely” ... to ... 5: “Do not trust at all”): *(i)* WWF; *(ii)* Doctors Without Borders; *(iii)* Amnesty International; *(iv)* SOS Kinderdorf; *(v)* Caritas; *(vi)* Licht ins Dunkel; *(vii)* Oxfam; *(viii)* Red Cross; *(ix)* *WHO* Covid-19 Response Fund.

(Q21) I think the organisation’s help is: (Answers ranging from 1: “Exclusively national” ... to ... 5: “Exclusively international”): *(i)* WWF; *(ii)* Doctors Without Borders; *(iii)* Amnesty International; *(iv)* SOS Kinderdorf; *(v)* Caritas; *(vi)* Licht ins Dunkel; *(vii)* Oxfam; *(viii)* Red Cross; *(ix)* *WHO* Covid-19 Response Fund.

(Q22) How relevant do you think is the work of the following organizations in relation to the Covid-19 disease?: (Answers ranging from 1: “Not at all relevant” ... to ... 5: “Very relevant”): *(i)* WWF; *(ii)* Doctors Without Borders; *(iii)* Amnesty International; *(iv)* SOS Kinderdorf; *(v)* Caritas; *(vi)* Licht ins Dunkel; *(vii)* Oxfam; *(viii)* Red Cross; *(ix)* *WHO* Covid-19 Response Fund.

C. Supplementary analysis

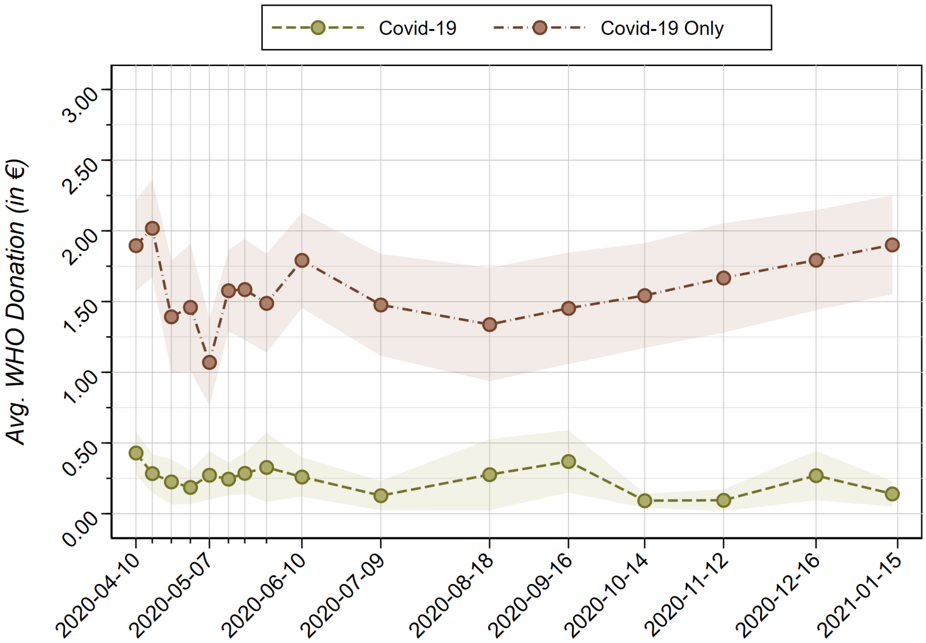

Figure S2: Observation 1: *avg. WHO donations* (in €) in *Covid-19* and *Covid-19 Only* over the eight consecutive weeks plus the eight consecutive months of data collection. Shaded areas indicate 95% confidence intervals. The differences (based on Tobit regressions of total donations on a treatment indicator, with €0 and €3 as the lower and upper limit, respectively, and robust standard errors) between treatments *Covid-19* and *Covid-19 Only* are statistically significant ($p < 0.001$) for all comparisons.

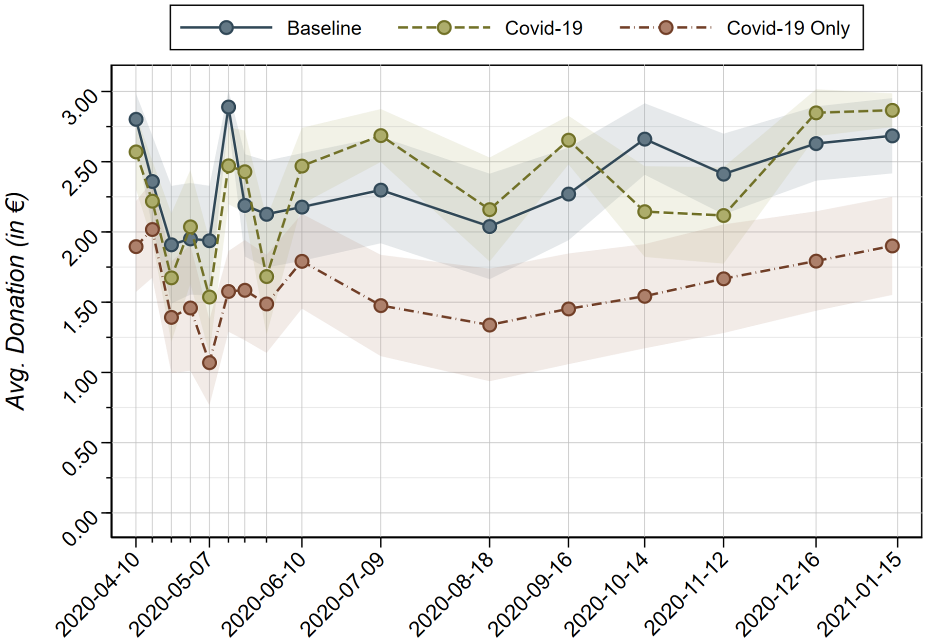

Figure S3: Observation 2: *avg. total* donations (in €) by treatments over the eight consecutive weeks plus the eight consecutive months of data collection. Shaded areas indicate 95% confidence intervals. The differences (based on Tobit regressions of total donations on a treatment indicator, with €0 and €3 as the lower and upper limit, respectively, and robust standard errors) between treatments *Baseline* and *Covid-19* are insignificant for each date, except for 2020-05-14 ($t(83) = 2.192$, $p = 0.031$) and 2020-10-14 ($t(73) = 2.909$, $p = 0.005$). The differences between treatments *Covid-19* and *Covid-19 Only* are statistically significant on the following dates: 2020-04-10 ($t(73) = 3.191$, $p = 0.002$), 2020-05-14 ($t(87) = 4.325$, $p < 0.001$), and 2020-05-20 ($t(68) = 3.307$, $p = 0.001$), 2020-06-10 ($t(75) = 2.952$, $p = 0.004$), 2020-07-09 ($t(64) = 4.946$, $p < 0.001$), 2020-08-18 ($t(65) = 2.524$, $p = 0.014$), 2020-09-16 ($t(70) = 4.369$, $p < 0.001$), 2020-12-16 ($t(76) = 4.320$, $p < 0.001$), and 2021-01-15 ($t(78) = 4.234$, $p < 0.001$).
